# Genomic characterization of a carbapenem-resistant *Leclercia adecarboxylata* isolate co-harboring *bla*_NDM-7_ and *bla*_KPC-4_ in a hospitalized patient

**DOI:** 10.1128/spectrum.01568-26

**Published:** 2026-09-29

**Authors:** Tracy H. Hazen, Gwen L. Robinson, Indira French, Sierra Spindler, Michael S. Humphrys, J. Kristie Johnson, Surbhi Leekha, L. Leigh Smith

**Affiliations:** 1 Center for Advanced Microbiome Research and Innovation, University of Maryland School of Medicine 12264, Baltimore, Maryland, USA; 2 Institute for Genome Sciences, University of Maryland School of Medicine 12264, Baltimore, Maryland, USA; 3 Department of Microbiology and Immunology, University of Maryland School of Medicine 12264, Baltimore, Maryland, USA; 4 Department of Epidemiology and Public Health, University of Maryland School of Medicine 12264, Baltimore, Maryland, USA; 5 Department of Pathology, University of Maryland School of Medicine 12264, Baltimore, Maryland, USA; University at Albany, Albany, New York, USA

**Keywords:** KPC, NDM, *Leclercia adecarboxylata*, carbapenem resistance

## Abstract

Carbapenems are a last-resort treatment for severe infections caused by multidrug-resistant organisms. Acquired carbapenemases located on plasmids can be harbored by frequent pathogens as well as opportunistic pathogens such as *Leclercia adecarboxylata*, which can colonize and cause infections in immunocompromised patients. In this study, we describe a carbapenem-resistant *L. adecarboxylata* rectal surveillance isolate La1358 from a hospitalized patient in Maryland in 2025. This isolate was obtained during routine rectal swab surveillance to detect drug-resistant organisms colonizing patients in intensive care units (ICUs). This is the first known description of an *L. adecarboxylata* isolate harboring both *bla*_KPC-4_ and *bla*_NDM-7_. The *bla*_KPC-4_ was identified on an IncFIA(HI1)/repB(R1701) plasmid and *bla*_NDM-7_ on an IncX3 plasmid. The *bla*_NDM-7_-containing IncX3 plasmid was nearly identical to a plasmid from an *Enterobacter hormaechei* isolate obtained in 2023 from another patient at the same hospital, demonstrating *L. adecarboxylata* is contributing to persistence of this plasmid among this patient population. These findings demonstrate that *L. adecarboxylata* colonizing patients can provide a reservoir of carbapenemases, including *bla*_NDM-7_, linking a less common species of Enterobacterales to the increasing prevalence of *bla*_NDM-7_ in the United States. Previous *L. adecarboxylata* isolates, from outside the United States, have been described harboring *bla*_NDM-1_; however, to our knowledge, this is the first description of *L. adecarboxylata* in the United States harboring *bla*_NDM-7_, acquired via a conserved IncX3 plasmid. Also, these findings highlight the importance of genomic surveillance to track plasmid-mediated dissemination of acquired carbapenemases among organisms colonizing hospitalized patients, in addition to isolates from clinical specimens associated with infection.

**IMPORTANCE:**

Carbapenem-resistant infections are a serious concern for hospitalized patients and have been increasing in the United States. This has been driven by acquired carbapenemases, in particular *bla*_KPC_ and *bla*_NDM_. A majority of acquired carbapenemases among hospitalized patients are harbored by frequent human pathogens; however, less common Enterobacterales can also acquire and disseminate plasmids harboring *bla*_KPC_ and *bla*_NDM_. This study describes the genome of a *bla*_KPC_ and *bla*_NDM_-containing *Leclercia adecarboxylata* isolate, an opportunistic human pathogen, identified colonizing a hospitalized patient. International *L. adecarboxylata* isolates have been described harboring *bla*_NDM-1_; however, to our knowledge, this represents the first description of *L. adecarboxylata* harboring *bla*_NDM-7_, which is a *bla*_NDM_ variant becoming more prevalent in the United States. These findings highlight the importance of genomic surveillance of drug-resistant organisms colonizing hospitalized patients to identify and disrupt reservoirs and transmission pathways linked to the spread and persistence of carbapenem resistance in hospitals.

## OBSERVATION

Carbapenem-resistant Enterobacterales (CRE) are an urgent concern to public health since carbapenems are a last-resort antibiotic treatment for severe infections (1). The Centers for Disease Control and Prevention (CDC) reported a 69% increase in the prevalence of CRE from 2019 to 2023 (2). This alarming increase was attributed to the continued spread of the *Klebsiella pneumoniae* carbapenemase (KPC) and a 461% increase in the New Delhi metallo-β-lactamase (NDM) (2). In the United States the most prevalent acquired carbapenemases have been KPC-2, KPC-3, and NDM-1 (3); however, there have been recent national and global increases in less prevalent variants such as NDM-5 and NDM-7 (4, 5).

Acquired carbapenemases are most often detected among human pathogens such as *Klebsiella pneumoniae, Escherichia coli,* and *Enterobacter cloacae* (2); however, they can be carried by diverse Enterobacterales, including species less often associated with human illness (6). *Leclercia adecarboxylata* is a member of the Enterobacterales found in soil, water, food, and associated with colonization of animals and the human gut (7–9). *L. adecarboxylata* has been linked to human infections, including urinary tract infection, wound infection, pneumonia, and bacteremia, but is most often associated with polymicrobial infections or infections in immunocompromised patients (7, 9). Carbapenem-resistant *L. adecarboxylata* isolates have been described carrying *bla*_NDM-1_ (10–12), *bla*_VIM-1_ (13), and *bla*_IMP_ (14); however, to our knowledge, there have been no published descriptions of isolates harboring *bla*_NDM-7_ and/or *bla*_KPC_.

In this study, we describe the genome of an *L. adecarboxylata* isolate containing the acquired carbapenemase genes, *bla*_NDM-7_ and *bla*_KPC-4_. Two carbapenem-resistant *L. adecarboxylata* isolates were obtained from a single patient admitted to an intensive care unit (ICU) in 2025. The patient was transferred from an outside hospital and underwent routine surveillance screening for carbapenem-resistant organisms by peri-rectal swab culture using selective media. Admission culture was negative; however, a repeat screening culture on day 4 of hospitalization grew carbapenem-resistant *L. adecarboxylata* (La1096). The patient was placed on rifaximin for hepatic encephalopathy and treated empirically for possible infection with five days of piperacillin-tazobactam. A subsequent peri-rectal swab on day 16 also grew carbapenem-resistant *L. adecarboxylata* (La1358). There were no additional *L. adecarboxylata* clinical or surveillance isolates from other patients while this patient was in the ICU. This *L. adecarboxylata* was considered acquired by the patient prior to ICU admission but not detected in the initial rectal surveillance culture.

Species identification was performed by VitekMS (Biomérieux). Antimicrobial susceptibility testing was performed by MicroScan (Beckman Coulter) and interpreted using CLSI breakpoints (15). The *L. adecarboxylata* isolates exhibited resistance to carbapenems, cephalosporins, and β-lactams and were susceptible to aminoglycosides, trimethoprim-sulfamethoxazole, tetracycline, and tigecycline (Table S1).

Draft genomes of La1096 and La1358 were generated by Illumina sequencing. Genomic DNA was extracted using the Mag-Bind Bacterial DNA 96 kit (Omega Bio-tek). Sequencing libraries were generated with NEBNext Ultra II DNA library kit (New England Biolabs) and sequenced as paired-end 150 bp on the Illumina Novaseq 6000. Illumina reads were assembled using SKESA v. 2.5.1 (16). Additional long-read sequence data was generated for La1358. Genomic DNA was extracted using the Nanobind CBB kit (PacBio). Libraries were generated using the Rapid Barcoding Kit (SQK-RBK114.24) and sequenced on a MinION MK1D (Oxford Nanopore Technologies). Basecalling was performed using the super accurate protocol (sup). Reads were assembled using Autocycler v.0.5.2 (17), which generated a resolved consensus assembly with a single circularized chromosome and four circularized plasmids. The genome assembly has 100% completeness and <1% determined using CheckM2 (18), and taxonomic classification was performed using GTDB-Tk v.2.5.2 (19). The chromosome has a sequence length of 4,940,622 bp and a G + C content of 55.63%, which is consistent with *L. adecarboxylata* genomes in GenBank.

Phylogenomic analysis was performed to compare the genome of La1358 to publicly available *L. adecarboxylata* genomes. There were 400 *L. adecarboxylata* genome or metagenome assemblies in GenBank as of April 2026; however, only 88 had an available RefSeq assembly with ≥97% completeness that passed NCBI taxonomy check as *L. adecarboxylata* and was not a different *Leclercia* spp. The RefSeq genomes and La1358 were compared to a reference (RefSeq accession no. GCF_020097395.1) using snippy v.4.6.0 (20) to identify single nucleotide polymorphisms (SNPs). A maximum-likelihood phylogeny was inferred from the core SNP alignment using model GTR + G + ASC with IQ-TREE 3 (21) and visualized using iTOL v.7 (22). Pairwise SNP distances were determined using snp-dists v.0.8.2 (https://github.com/tseemann/snp-dists). Antimicrobial resistance genes were identified using AMRFinderPlus v.4.0.23 (23). Plasmids were characterized using PlasmidFinder v.2023-01-18 (24) and Mob-suite v.3.1.9 (25). The genomes were annotated using Prokka v.1.14.6 (26). Plasmid maps were generated using Circos v.0.69-10 (27) and diagrams using EasyFig (28).

**Fig 1:**
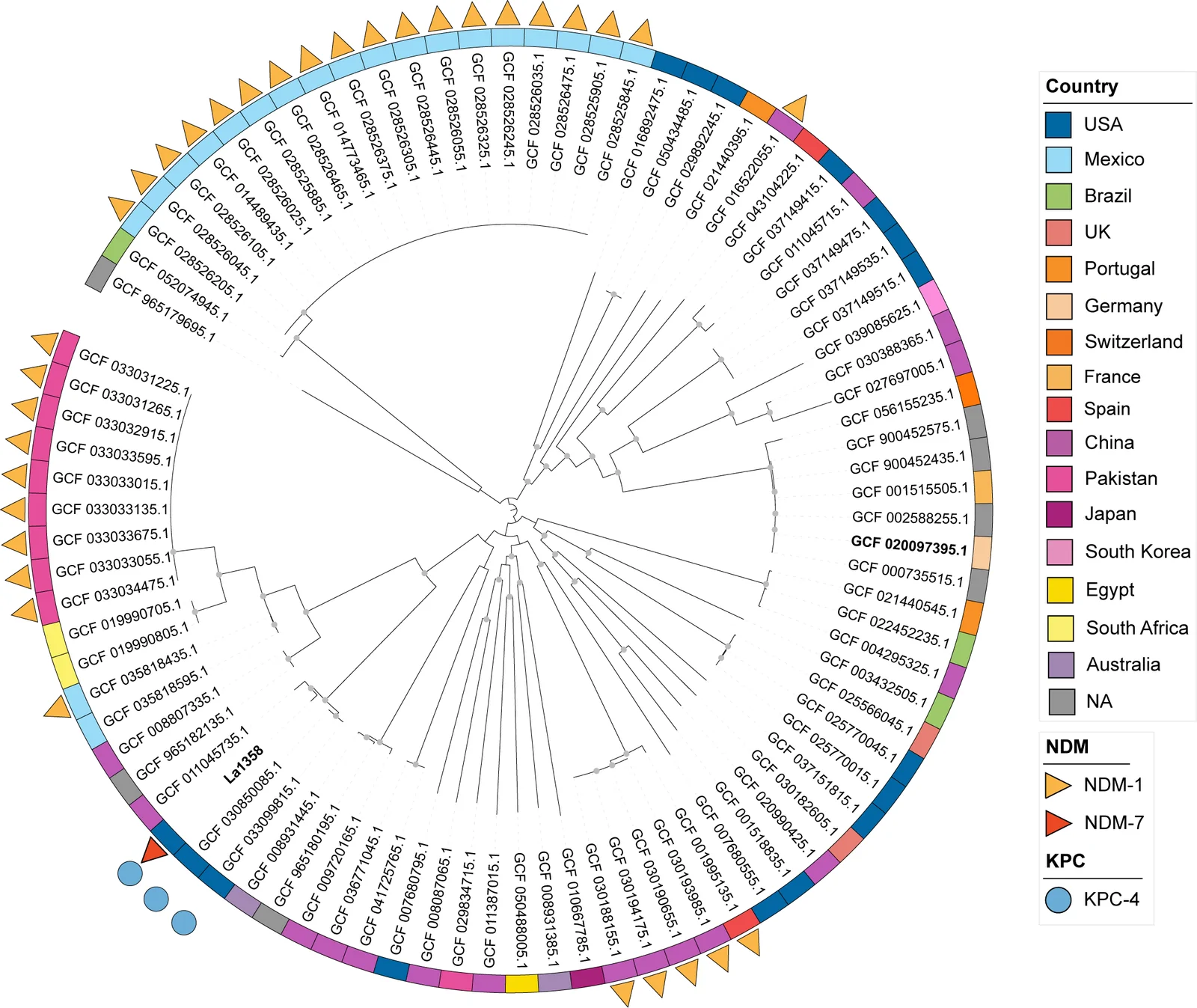
Phylogenomic analysis of La1358. A maximum-likelihood phylogeny examining the genetic relatedness of La1358 to previously described *L. adecarboxylata* genomes available in RefSeq. The country of isolation is indicated with different colors (see inset figure legend) or not available (NA). A triangle indicates genomes containing a *bla*_NDM_, allele while a circle denotes a *bla*_KPC_.

There is no available multilocus sequence typing scheme for *L. adecarboxylata,* therefore phylogenomic analysis was used to compare La1358 to previously characterized genomes of *L. adecarboxylata* from RefSeq (Fig. 1). La1358 was most similar to a blood isolate from China in 2017 (RefSeq accession no. GCF_011045735.1) and two isolates from a hospital environment in Minnesota from 2023 (RefSeq accession nos. GCF_030850085.1 and GCF_033099815.1). However, La1358 differed from these isolates by 1,569–2,301 SNPs, indicating it is a different strain. A *bla*_KPC_ gene, *bla*_KPC-4_, was identified in the Minnesota hospital isolates and La1358. A *bla*_NDM_ gene, all *bla*_NDM-1_, was identified in 38% (34/88) of the previously characterized *L. adecarboxylata*. The *bla*_NDM-1_-containing isolates originated from outside the United States and were associated with an outbreak in Mexico (29), a hospital environment in Pakistan, and broiler farms in China (30).

**Fig 2:**
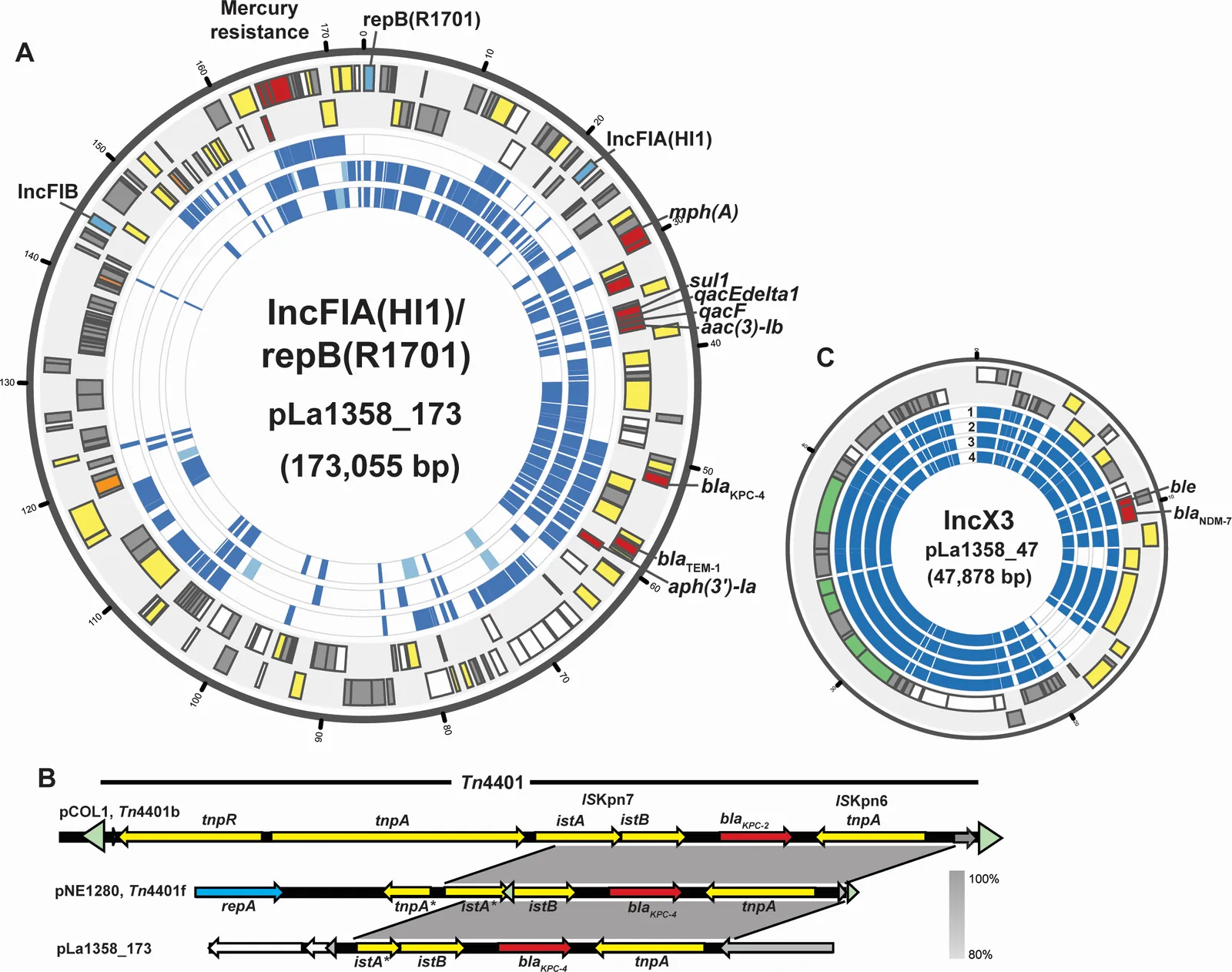
Comparison of the La1358 carbapenemase-encoding plasmids to plasmids of other Enterobacterales. Genes are color-coded by function as follows: resistance (red), replication and partitioning (blue), toxins (orange), mobile elements (yellow), conjugation (green), hypothetical (gray), and other (white). (**A**) Plasmid map with predicted protein-coding genes of the 173 kb *bla*_KPC-4_-containing IncFIA(HI1)/repB(R1701) hybrid plasmid pLa1358_173. The inner tracks indicate the similarity of pLa1358_173 genes in other *bla*_KPC-4_-containing Enterobacterales isolates: *L. adecarboxylata* from the environment of a Minnesota hospital (RefSeq accession no. GCF_030850085.1) (outer data track), *Enterobacter hormaechei* isolate Eh1166 (middle data track), and *Enterobacter intestinihominis* isolate Eint1770 (inner data track) from the same hospital as La1358 from additional patients at the same hospital as La1358. Blue indicates genes with ≥80% nucleotide identity and ≥60% alignment length. (**B**) The *bla*_KPC-4_ genetic environment of pLa1358_173 compared with the previously described *bla*_KPC-4_ located within *Tn*4401f on plasmid pNE1280 (GenBank acc. no. JQ837276.1) (33), and the *bla*_KPC-2_-containing *Tn*4401b from pCOL1 (GenBank: EU176013.1). The *Tn*4401 region is located between light green triangles representing the inverted repeats. An asterisk indicates pseudogenes that are truncated and/or not protein-coding. The *istA* of pLa1358_173 is similarly truncated; however, the partial *tnpA* is missing. (**C**) Plasmid map with the predicted protein-coding genes of the *bla*_NDM-7_-containing IncX3 plasmid from *L. adecarboxylata* La1358. Inner tracks indicate similarity to *bla*_NDM-7_-containing IncX3 plasmids from other Enterobacterales including: *E. hormaechei* isolate Eh142 from the same hospital as La1358 (track 1), an *E. cloacae* from France (RefSeq accession no. GCF_050031195.1) (track 2), *K. pneumoniae* from China (GenBank accession no. CP025215.1) (track 3), and an *E. coli* from China (MH523639.1) (track 4). Blue indicates genes with ≥96% nucleotide identity and ≥60% alignment length.

The *bla*_KPC-4_ was identified on a 173-kb IncFIA(HI1)/repB(R1701) hybrid plasmid that also contains *bla*_TEM-1_, *mph(A), sul1*, *qacEdelta1, qacF, aac*(3)-Ib, and *aph*(3')-Ia (Fig. 2A). These genes can confer resistance to penicillins, macrolides, sulfonamides, disinfectants, and aminoglycosides. IncFIA plasmids have been described carrying *bla*_KPC-2_ and *bla*_KPC-3_, and repB(R1701) plasmids have hybridized with IncFIA(HI1) and other replicons, including IncFIB(K) and IncFII(K) (31, 32). The *bla*_KPC-4_ gene was previously identified in a truncated *Tn*4401 element designated *Tn*4401f inserted in an IncL/M plasmid (33). The genetic region of *bla*_KPC-4_ of La1358 is partially truncated compared with that of *Tn*4401f, which may have occurred as this region was acquired by the IncFIA(HI1)/repB(R1701) plasmid (Fig. 2B). Comparison of pLa1358_173 to the *bla*_KPC-4_-containing *L. adecarboxylata* isolate from a Minnesota hospital and two *bla*_KPC-4_-containing *Enterobacter* spp. isolates that were also obtained from the same hospital as La1358 in the same year (2025) demonstrated limited plasmid similarity, suggesting independent acquisition of the *bla*_KPC-4_-containing plasmids (Fig. 2A).

The *bla*_NDM-7_ gene was identified on a 47.8-kb IncX3 plasmid, which has been linked to the spread of *bla*_NDM_ among Enterobacterales (34). The *bla*_NDM-7_-containing IncX3 plasmid from La1358 was similar to a *bla*_NDM-7_-containing IncX3 plasmid from an *E. hormaechei* isolate obtained in 2023 from the same hospital, as well as previously characterized *E. cloacae, K. pneumoniae,* and *E. coli* from France and China with 97%–100% nucleotide identity over >90% of its length (Fig. 2C). This suggests La1358 acquired a conserved IncX3 plasmid involved in the dissemination of *bla*_NDM-7_ among different species of Enterobacterales in the U.S. and internationally.

In conclusion, our findings demonstrate that *L. adecarboxylata* colonizing hospitalized patients in the United States can co-harbor multiple acquired carbapenemases. This is an important finding linking less common species of Enterobacterales infrequently associated with infections to the increasing prevalence of *bla*_NDM-7_ in the United States. These findings highlight the importance of genomic surveillance to track carbapenem resistance and other transmissible drug resistance mechanisms among organisms colonizing hospitalized patients.

## Data Availability

Sequence reads and assemblies have been deposited under BioProject PRJNA1422675.

## SUPPLEMENTAL MATERIAL

Table S1 spectrum.01568-26-s0001.xlsx Antimicrobial susceptibilities of the carbapenem-resistant *L. adecarboxylata* human fecal isolates.

ASM does not own the copyrights to Supplemental Material that may be linked to, or accessed through, an article. The authors have granted ASM a non-exclusive, world-wide license to publish the Supplemental Material files. Please contact the corresponding author directly for reuse.
